# Effect of vitamin A on the relationship between maternal thyroid hormones in early pregnancy and fetal growth: A prospective cohort study

**DOI:** 10.3389/fnut.2022.980853

**Published:** 2022-08-24

**Authors:** Yanyu Lyu, Qingyong Xiu, Hanxiao Zuo, Guangfei Xu, Xiaodai Cui, Zhenfeng Sun, Rong Mi, Lijun Wu

**Affiliations:** ^1^Experiment Center, Capital Institute of Pediatrics, Beijing, China; ^2^Department of Pediatrics, Beijing Daxing Maternal and Child Care Hospital, Beijing, China; ^3^School of Public Health, University of Alberta, Edmonton, AB, Canada; ^4^Department of Nutrition, School of Public Health, Nantong University, Nantong, China; ^5^Department of Obstetrics, Beijing Daxing Maternal and Child Care Hospital, Beijing, China; ^6^Department of Neonatology, Children's Hospital of Capital Institute of Pediatrics, Beijing, China; ^7^Department of Epidemiology, Capital Institute of Pediatrics, Beijing, China

**Keywords:** thyroid hormones, vitamin A, birth weight, small size for gestational age, large size for gestational age

## Abstract

**Background:**

Fetal growth patterns are influenced by maternal thyroid function and vitamin A level during pregnancy. Vitamin A presents interactions with thyroid tissues and hormonal systems. We examined whether vitamin A status modified the associations of maternal thyroid hormones in early pregnancy and fetal growth outcomes among euthyroid pregnant women in a prospective cohort study (*n* = 637).

**Methods:**

We performed multiple linear regression and multinomial logistic regression analysis to investigate the effects of thyroid hormones in early pregnancy on fetal growth according to different levels of serum vitamin A based on median value.

**Results:**

A 1 pmol/L increase in maternal free triiodothyronine (FT3) levels was associated with an increased birth weight of 0.080 kg (*p* = 0.023) in women with lower maternal vitamin A levels in early pregnancy. Increased maternal free thyroxine (FT4) was associated with decreased odds for both small size for gestational age (SGA) [odds ratios (OR) = 0.66, 95% confidence interval (CI): 0.45–0.95] and large size for gestational age (LGA) (OR = 0.66, 95% CI: 0.45–0.98) in women with higher vitamin A level in early pregnancy after adjustment for maternal prepregnancy body mass index, gestational weight gain, maternal employed, parity, gestational week at sampling, and gestational diabetes mellitus.

**Conclusions:**

In Chinese pregnant women without overt thyroid dysfunction, maternal FT4 in early pregnancy was positively associated with optimal fetal growth among women with higher serum vitamin A concentrations.

## Introduction

Thyroid hormones have multiple fundamental physiological functions and are essential for vertebrate embryogenesis and fetal maturation (1). The human fetal thyroid gland synthesizes thyroid hormones after 12 weeks of gestation, and the fetus completely depends on maternal supply in the first trimester (2, 3).

Well-established adverse birth outcomes for overt hypothyroidism and hyperthyroidism during pregnancy included preterm delivery, low birth weight, and fetal growth restriction (4–6). Subclinical thyroid dysfunction is much more frequent than overt thyroid disease (7). However, the effect of maternal subclinical thyroid dysfunction during pregnancy on fetal growth remains controversial. A meta-analysis showed that maternal subclinical hypothyroidism in pregnancy is associated with a higher risk of small size for gestational age (SGA) and lower birth weight, whereas isolated hypothyroxinemia is associated with a lower risk of SGA and higher birth weight (8). A large population-based cohort study reported that maternal subclinical hypothyroidism before conception or in early pregnancy was associated with increased odds for large size for gestational age (LGA) in male newborns (9). Among euthyroid pregnant women, there also remain inconsistencies in the literature about the effects of thyroid hormones on fetal growth. A meta-analysis reported that there was an inverse, dose-response associations of maternal thyroid stimulating hormone (TSH) and free thyroxine (FT4) in the normal range with birth weight, with a higher effect estimate for measurement in the third trimester than in the first or second trimester (8). Persistently low FT4 concentrations throughout pregnancy increased the risk of LGA (10). Medici et al. (11) showed that maternal high-normal FT4 levels in early pregnancy increased the risk of SGA. However, a study in healthy pregnant women reported that lower FT4 and higher TSH levels during the first trimester were not associated with birth weight or SGA (12).

Vitamin A is a lipophilic micronutrient and is critical for cell proliferation and differentiation, immune, reproduction, embryonic development, and metabolism (13, 14). It has been identified that vitamin A interacts with endocrine tissues and hormones. Retinoids interfere with iodine metabolism in the thyroid and vitamin A deficiency aggravates thyroid dysfunction with a reduction of iodine uptake and thyroid hormones synthesis (15). Vitamin A and its metabolites such as retinoic acid (RA) can also regulate the effects of thyroid hormones on target tissues. There was a well-documented crosstalk between RA signaling and thyroid hormone signaling, and RA receptor (RAR) and thyroid hormone receptor (TR) modulate gene transcription through common hormone response elements (16). In terms of internal transport, vitamin A and thyroid hormones are linked by transthyretin (TTR), which is a tetrameric transport protein that transports thyroid hormone thyroxine (T4) and retinol-binding protein (RBP4) bounding to retinol (vitamin A) to form a ternary retinol-RBP4-TTR complex (17). TTR is not only an important hepatically derived protein carrier of thyroid hormones and retinol in blood, but also seems to play an important role in the delivery of maternal thyroid hormone to the fetus because of the synthesis, secretion, and uptake of TTR by the human placenta. High TTR concentrations near the maternal placental interface may bind maternal T4 for transporting to capillaries of the fetus (18).

Given that vitamin A may affect thyroid hormones synthesis and modulate the thyroid hormonal systems, we aimed to investigate whether circulating levels of vitamin A modifies the associations between maternal thyroid hormones in early pregnancy and fetal growth among women without overt thyroid dysfunction.

## Methods

### Study design and study population

In this prospective cohort study, pregnant women were approached by trained research assistants in the Prenatal Health Care Clinic in Beijing, China. They were eligible if they (i) were <13 gestational weeks; (ii) were > 18 years of age; (iii) planned to give birth and receive health care for their infants at our hospital; and (iv) consented to being followed up for at least 2 years postnatally. Staff involved in this project received specialized training by project investigators for interview, measurement and biospecimen collection and processing. The participants completed the first questionnaire, physical measurements, and blood sample collection at enrollment. They were then followed up in conjunction with their routine perinatal care, and all participants completed a questionnaire and biophysical measurements in the mid (24–28 weeks of gestation) and late (32–36 weeks of gestation) pregnancy. At birth, cord blood was collected, and the newborn's anthropometric indices were measured by trained medical staff. After birth, the mother and baby were followed up at 42 days, 3, 6, 9, and 12 months of age by pediatricians from Children's Hospital of Capital Institute of Pediatrics. Women who underwent *in vitro* fertilization, had a twin pregnancy or stillbirth, and had a history of thyroid disease or thyroid medication use during pregnancy were excluded. This prospective cohort study was approved by the Ethical Committee of Capital Institute of Pediatrics (SHERLL-2016034), and written informed consent was obtained from each subject before recruitment.

### Exposure variables

Thyroid hormones were measured at enrollment in early pregnancy for all participants. Serum triiodothyronine (T3) (reference range, 1.34 to 2.73 nmol/L), T4 (reference range, 78.38 to 157.4 nmol/L), TSH (reference range, 0.34 to 5.60 mIU/L), free triiodothyronine (FT3) (reference range, 3.8 to 6.0 pmol/L) and FT4 (reference range, 7.86 to 14.41 pmol/L) concentrations were measured using an Access Immunoanalyzer (Beckman Coulter Inc., Fullerton, CA) according to the manufacturer's protocol.

### Outcomes and covariables

The primary outcomes were birth weight, SGA and LGA. Infants were classified as SGA or LGA if their birth weight was lower than the 10^th^ percentile or higher than the 90^th^ percentile for gestational age according to the Fenton growth chart (19).

Potential confounders or risk factors included maternal variables such as gestational week at sampling, prepregnancy body mass index (BMI), parity, employed, gestational weight gain (GWG), and gestational diabetes mellitus (GDM). Infant variables including sex, gestational age at birth were also identified as risk factors. The prepregnancy BMI was calculated through self-reported prepregnancy weight and height measured at enrollment. Based on WHO-Asian criteria, women were classified as underweight (BMI <18.5 kg/m^2^), normal weight (18.5 to 23.9 kg/m^2^), overweight (24.0 to 27.9 kg/m^2^) and obesity (BMI ≥28.0 kg/m^2^) (20). GWG was calculated as the difference between body weight at birth and self-reported prepregnancy weight. All women were screened for GDM with a 75-g oral glucose tolerance test (OGTT) between 24 and 28 weeks of gestation as part of routine care. Venous blood samples were collected at 0, 1, and 2 h after a 75-g glucose load. If one or more of the blood glucose levels were met or exceeded the predefined levels (0 h (fasting) ≥ 5.10 mmol/L; 1 h ≥ 10.00 mmol/L; and 2 h ≥ 8.50 mmol/L), then women were diagnosed with GDM according to the recommendations of the International Association of the Diabetes and Pregnancy Study Groups Consensus Panel (21). Gestational age was determined as the best estimate according to the hierarchy of first trimester ultrasound, last menstrual period, and obstetric estimate.

### Modifier

Vitamin A (retinol) were measured at enrollment in early pregnancy for all participants. Serum was analyzed for retinol using high performance liquid chromatography (HPLC, Agilent, USA). The standard substance was purchased from the American Sigma Company. The Westgard multi-rule quality control procedure was adopted to analyze whether an analytical run was in-control.

### Statistical analysis

Characteristics of the study population were summarized using descriptive statistical methods. Continuous data were summarized as means (standard deviation, SD) or medians (interquartile ranges, IQR), and categorical data were presented as percentages. We examined medians and interquartile ranges for each of the thyroid hormones and vitamin A markers, and assessed crude differences among SGA, average size for gestational age (AGA) and LGA by the non-parametric test. To create continuous maternal thyroid hormones and vitamin A concentrations as exposure variables in the multivariate analysis, we log-transformed raw values if distributions were skewed after removing outliers (±4 SD from the mean) (8). We did not impute the missing data on maternal education for 4 (0.6%) pregnant women. The 4 pregnant women were ruled out automatically when running the regression that included the covariate of maternal education. Multiple linear regression models were conducted to explore the associations of maternal thyroid hormones and vitamin A levels with infant birth weight. We tested for effect modification of the associations of thyroid hormones with neonatal birth weight by vitamin A level as a continuous variable using a product interaction term of vitamin A concentration and each maternal thyroid hormone concentration. We further examined the potentially relevant differences by performing stratified analyses by lower and higher vitamin A levels based on the median parameter (median = 0.46) if there was any indication of effect modification. A *p*-value for interaction of <0.15 was used as a cutoff to further explore potentially relevant effect modification by stratification (22). The first model (model 1) included gestational age at sampling, fetal sex and gestational age at birth, and the quadratic form of gestational age at birth to account for the nonlinear relationship between pregnancy duration and birth weight. In the second model (model 2), we additionally adjusted for maternal prepregnancy BMI, parity, employed, GWG, and GDM. These factors were associated with birth weight identified using stepwise variable selection procedures.

A similar strategy was used to assess whether maternal thyroid hormones were associated with the risk of SGA or LGA and to test for effect modification by vitamin A concentrations. We carried out multinomial logistic regression analysis to estimate adjusted odds ratios (ORs) across the three categories of birth weight in the study population after accounting for important confounders (gestational age at sampling, maternal prepregnancy BMI, parity, employed, GWG and GDM). Since most of the interaction terms with vitamin A were significantly statistical, further analyses were conducted separately for low and high vitamin A levels to investigate the effect modification of vitamin A.

All statistical analyses were performed using R statistical software version 3.5.1 (R Project for Statistical Computing; http://www.r-project.org). Statistical significance was set at *p* < 0.05.

## Results

**Figure 1:**
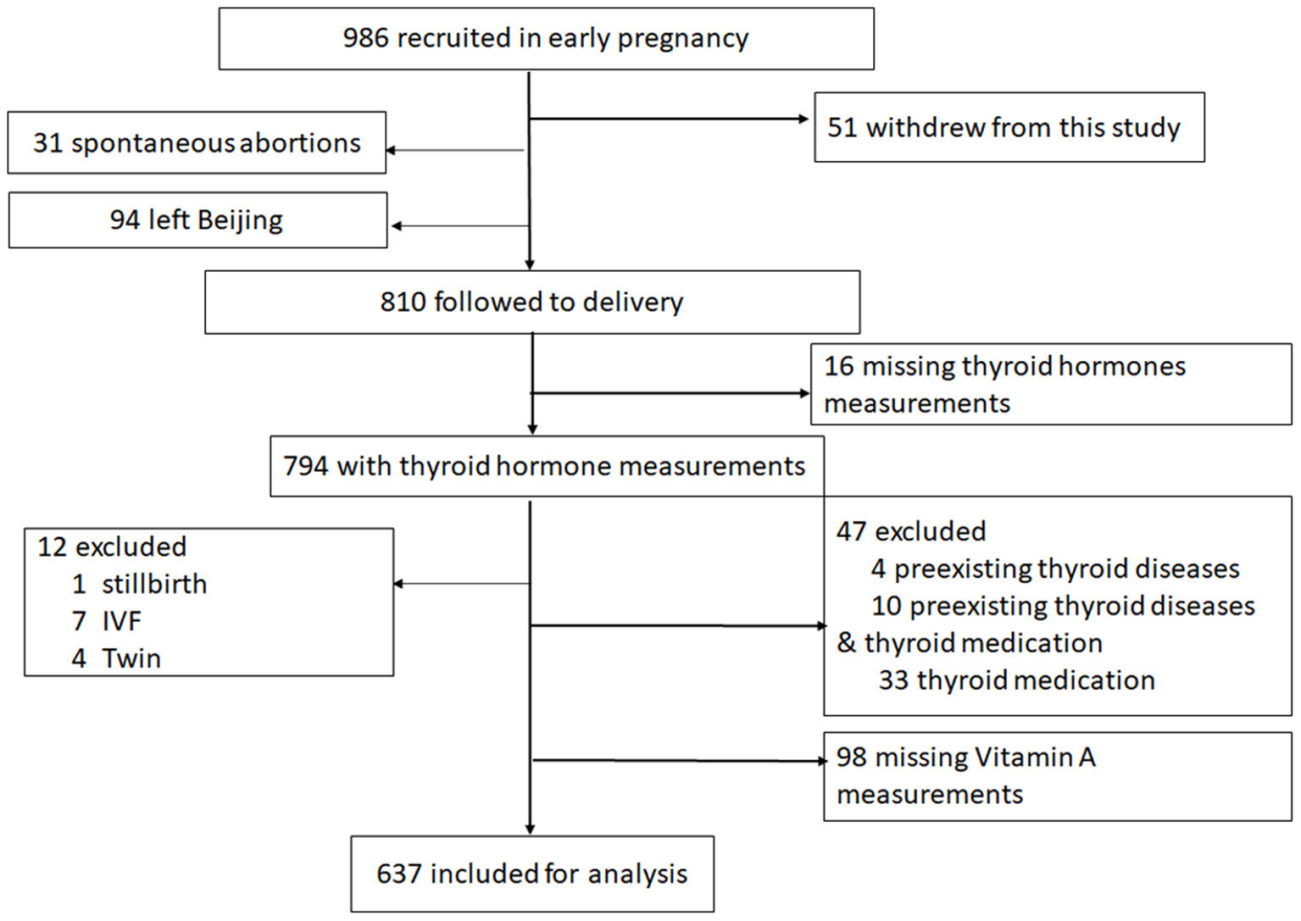
Flow diagram of study population.

The flow diagram of the study population is shown in Figure 1. A total of 986 pregnant women were approached and recruited before 13 weeks of gestation from November 2016 through December 2017. During the follow-up phase, 51 women declined to continue. Among the 935 women, 94 women left Beijing or returned to hometown to give birth, and 31 had spontaneous abortions. 810 women were followed to delivery, of which 16 women (1.98%) with missing thyroid hormone measurements in early pregnancy were excluded. Twelve women (1.48%) were excluded owing to the presence of stillbirth (*n* = 1), *in vitro* fertilization (IVF) (*n* = 7) or delivering twins (*n* = 4). Forty-seven women (5.80%) were excluded with preexisting thyroid diseases (*n* = 4), preexisting thyroid diseases and thyroid medication (*n* = 10) or thyroid medication (*n* = 33) including clinically overt hyperthyroidism, hypothyroidism, and Hashimoto's thyroiditis. Additionally, 98 women (12.10%) with missing vitamin A measurements were excluded. The remaining 637 women and their infants were included in this analysis, and their characteristics are described in Table 1.

**Table 1:** Distribution of maternal and fetal characteristics in study population (*n* = 637).

	**Mean ±SD or *N* (%)**
**Maternal characteristic**	
Age (year)	30.01 ± 3.77
Education^*^	
Middle-school or less	74 (11.69)
High school, vocational high school, or technical school	166 (26.22)
College	188 (29.70)
University graduate or more	205 (32.39)
Ethnicity—Han	611 (95.92)
Employed–Yes	418 (65.62)
Gestational age at sampling (week)	9.29 ± 1.73
Pre-pregnancy BMI (kg/m^2^)	22.56 ± 3.68
Underweight (BMI <18.5)	66 (10.36)
Normal (BMI 18.5 - <24)	380 (59.65)
Overweight (BMI 24 - <28)	134 (21.04)
Obese (BMI ≥ 28)	57 (8.95)
Nulliparity	334 (52.43)
Gestational diabetes mellitus	82 (12.87)
Gestational weight gain during pregnancy (kg)	14.54 ± 4.57
**Infant characteristics**	
Birth weight (g)	3,374 ± 411
Gestational age at birth (week)	39.47 ± 1.17
SGA	37 (5.81)
LGA	30 (4.71)
Male	321 (50.39)

* Missing information: 4 for maternal education.

SD, standard deviation; IQR, interquartile range; BMI, body mass index; SGA, small size for gestational age; LGA large size for gestational age.

Table 2 compares medians and interquartile ranges of the five thyroid hormones and vitamin A concentrations across the three categories of birth weight. The levels of FT4 were significantly different among the SGA, AGA, and LGA groups (*p* < 0.05). FT4 was the highest in AGA infants, with a concentration of 10.94 pmol/L, and the interquartile range was from 10.04 to 11.91. T4 showed a trend of increase in the SGA, AGA, and LGA groups, although the difference was marginally significant (*p* = 0.080). We assessed correlations among thyroid hormones and vitamin A, and vitamin A was correlated with these thyroid hormones except for T4 (see Supplementary Table 1).

**Table 2:** Distribution of maternal thyroid hormones and vitamin A levels in early pregnancy among SGA, AGA, and LGA newborns.

	**Total (*n* = 637)**	**SGA (*n* = 37)**	**AGA (*n* = 570)**	**LGA (*n* = 30)**	*p*-value^*^
	**Median (IQR)**	**Median (IQR)**	**Median (IQR)**	**Median (IQR)**	
T3 (nmol/L)	1.90 (1.67–2.16)	1.89 (1.71–2.07)	1.90 (1.65–2.17)	1.90 (1.73–2.09)	0.882
T4 (nmol/L)	129.20 (114.07–144.86)	123.26 (112.16–135.16)	129.20 (114.12–144.86)	136.02 (123.02–151.43)	0.080
TSH (mIU/L)	1.23 (0.72–1.81)	1.22 (0.84–1.93)	1.23 (0.71–1.80)	1.42 (0.89–1.92)	0.611
FT3 (pmol/L)	4.97 (4.68–5.41)	4.94 (4.65–5.45)	4.98 (4.69–5.40)	4.92 (4.66–5.41)	0.967
FT4 (pmol/L)	10.91 (9.94–11.89)	10.35 (9.41–11.56)	10.94 (10.04–11.91)	10.34 (9.29–11.58)	**0.046**
Vitamin A (mg/L)	0.46 (0.41–0.53)	0.45 (0.42–0.54)	0.46 (0.41–0.53)	0.50 (0.42–0.58)	0.598

* kruskal-Wallis test.

SGA, small size for gestational age; AGA, average size for gestational age; LGA, large size for gestational age; IQR, interquartile range; T3, triiodothyronine; T4, thyroxin; TSH, thyroid stimulating hormone; FT3, free triiodothyronine; FT4, free thyroxine.

Those highlighted in bold indicate that the associations showed statistical significance.

The associations of thyroid hormones and vitamin A with infant birth weight are presented separately in Table 3. We found that FT3 was associated with birth weight when modeled jointly with vitamin A. In model 1 (corrected for gestational age at sampling, gestational age at birth, and fetal sex), the birth weight increased by 0.245 kg [95% confidence interval (CI), −0.027 to 0.517] for a 1 pmol/L increase in FT3 levels. After complete adjustment, this relationship became marginally significant [regression coefficient (b), 0.265, 95% CI, −0.001 to 0.531].

**Table 3:** Adjusted linear associations of maternal thyroid hormones and vitamin A level in early pregnancy and infant birth weight, displayed as single effects and as joint effects.

	**Model 1^a^**		**Model 2^b^**	
	**β (95% CI)**	***p*-value**	**β (95% CI)**	***p*-value**
T3 (nmol/L)	0.114 (−0.248–0.476)	0.538	0.063 (−0.292–0.417)	0.730
Vitamin A (mg/L)	0.559 (−0.92–2.038)	0.459	0.078 (−1.378–1.535)	0.916
T3* vitamin A^c^	−0.190 (−0.941–0.561)	0.620	−0.063 (−0.799–0.672)	0.866
T4 (nmol/L)	0.002 (−0.005–0.008)	0.635	0.001 (−0.005–0.007)	0.705
Vitamin A (mg/L)	0.137 (−1.597–1.871)	0.877	−0.253 (−1.951–1.445)	0.770
T4* vitamin A^c^	0.001 (−0.013–0.014)	0.937	0.002 (−0.011–0.015)	0.797
TSH, log	−0.114 (−0.278–0.05)	0.175	−0.112 (−0.272–0.049)	0.175
Vitamin A (mg/L)	0.207 (−0.095–0.508)	0.180	−0.022 (−0.332–0.287)	0.887
TSH* vitamin A^c^	0.199 (−0.157–0.556)	0.274	0.182 (−0.167–0.531)	0.307
FT3 (pmol/L)	0.245 (−0.027–0.517)	0.078	0.265 (−0.001–0.531)	0.051
Vitamin A (mg/L)	2.465 (−0.39–5.319)	0.091	2.468 (−0.328–5.263)	0.084
FT3* vitamin A^c^	−0.453 (−1.021–0.115)	**0.118**	−0.499 (−1.056–0.057)	**0.079**
FT4 (pmol/L)	0.030 (−0.042–0.103)	0.414	0.046 (−0.025–0.118)	0.205
Vitamin A (mg/L)	0.799 (−0.875–2.473)	0.350	0.768 (−0.874–2.41)	0.360
FT4* vitamin A^c^	−0.053 (−0.207–0.101)	0.501	−0.07 (−0.222–0.081)	0.362

a Model 1: Adjusted for gestational week at sampling, fetal sex, and gestational age at birth (linear and quadratic).

b Model 2: Adjusted for model 1 plus maternal pre-pregnancy BMI, parity, employed, gestational weight gain, gestational diabetes mellitus.

c The coefficient of the interaction term in regression model.

CI, confidence interval; T3, triiodothyronine; T4, thyroxin; TSH, thyroid stimulating hormone; FT3, free triiodothyronine; FT4, free thyroxine.

Those highlighted in bold indicate that the associations showed statistical significance.

We conducted further analyses separately for vitamin A levels by median 0.46 mg/L to investigate the effect of vitamin A differences. Table 4 shows that FT3 levels were significantly associated with infant birth weight in the lower vitamin A levels group (*p* = 0.023). The infant birth weight increased by 0.080 kg (95% CI, 0.012 to 0.147) for a 1 pmol/L increase in FT3 levels when the mother was in low vitamin A status in early pregnancy.

**Table 4:** Associations between maternal FT3 and infant birth weight, stratified by vitamin A level.

**FT3**	**Lower vitamin A level (*n* = 319)**		**Higher vitamin A level (*n* = 318)**	
	**β (95% CI)**	***p*-value**	**β (95% CI)**	***p*-value**
Model 1^a^	0.086 (0.017–0.156)	**0.015**	−0.022 (−0.097–0.053)	0.561
Model 2^b^	0.080 (0.012–0.147)	**0.023**	−0.036 (−0.11–0.038)	0.343

a Model 1: Adjusted for gestational week at sampling, fetal sex, and gestational age at birth (linear and quadratic).

b Model 2: Adjusted for model 1 plus maternal pre-pregnancy BMI, parity, employed, gestational weight gain, gestational diabetes mellitus.

FT3, free triiodothyronine; CI, confidence interval.

Those highlighted in bold indicate that the associations showed statistical significance.

Stratified analyses by lower and higher vitamin A levels based on the median parameter (median = 0.46 mg/L).

As shown in Table 5, T4, TSH (log transformed), FT3 and FT4 were associated with LGA when modeled jointly (i.e., interaction) with vitamin A. The interaction term of FT3 and vitamin A and the interaction term of FT4 and vitamin A were both associated with SGA (*p* < 0.15). In the stratified analysis, increased levels of FT4 were associated with a significantly reduced risk of both SGA and LGA in the high vitamin A levels group after full adjustment (OR, 0.66, 95% CI: 0.45 to 0.95 for SGA; OR, 0.66, 95% CI: 0.45 to 0.98 for LGA) in the multinomial logistic regression analyses.

**Table 5:** Associations between maternal thyroid hormones in early pregnancy and odds of SGA or LGA infants in multinomial logistic regression models, stratified by vitamin A level.

	**Model 1^a^**					**Model 2^b^**				
	**Lower vitamin A level**		**Higher vitamin A level**		***p* for interaction**	**Lower vitamin A level**		**Higher vitamin A level**		***p* for interaction**
	**(*n* = 319)**		**(*n* = 318)**			**(*n* = 319)**		**(*n* = 318)**		
	**OR (95% CI)**	***p*-value**	**OR (95% CI)**	***p*-value**		**OR (95% CI)**	***p*-value**	**OR (95% CI)**	***p*-value**	
**T3**										
SGA	0.76 (0.22–2.66)	0.668	1.80 (0.54–6.05)	0.341	0.189	0.60 (0.15–2.48)	0.483	1.47 (0.43–5.08)	0.543	0.322
LGA	1.07 (0.27–4.17)	0.928	0.40 (0.09–1.81)	0.235	0.408	0.85 (0.19–3.91)	0.835	0.31 (0.06–1.69)	0.178	0.497
**T4**										
SGA	0.99 (0.97–1.01)	0.264	0.98 (0.96–1.01)	0.156	0.835	0.99 (0.97–1.01)	0.324	0.98 (0.96–1.01)	0.168	0.848
LGA	1.02 (1.00–1.04)	0.135	0.99 (0.97–1.01)	0.392	**0.110**	1.02 (1.00–1.05)	0.080	0.99 (0.97–1.02)	0.477	**0.108**
**Log (TSH)**										
SGA	1.22 (0.72–2.08)	0.464	1.22 (0.63–2.36)	0.564	0.950	1.21 (0.68–2.17)	0.521	1.16 (0.60–2.26)	0.658	0.983
LGA	1.00 (0.58–1.77)	0.979	2.03 (0.85–4.83)	0.109	**0.066**	0.89 (0.49–1.62)	0.708	2.15 (0.84–5.47)	0.109	**0.059**
**FT3**										
SGA	0.60 (0.25–1.44)	0.252	1.54 (0.66–3.58)	0.314	**0.142**	0.41 (0.15–1.12)	0.082	1.58 (0.64–3.90)	0.326	**0.146**
LGA	1.50 (0.65–3.51)	0.345	0.62 (0.23–1.66)	0.340	**0.123**	1.30 (0.51–3.35)	0.585	0.44 (0.14–1.40)	0.162	**0.099**
**FT4**										
SGA	1.02 (0.80–1.30)	0.857	0.69 (0.49–0.98)	**0.036**	**0.111**	1.03 (0.77–1.36)	0.857	0.66 (0.45–0.95)	**0.025**	**0.060**
LGA	0.96 (0.70–1.30)	0.774	0.70 (0.49–1.00)	0.052	**0.089**	1.02 (0.74–1.42)	0.892	0.66 (0.45–0.98)	**0.040**	**0.059**

a Model 1: Adjusted for gestational week at sampling.

b Model 2: Adjusted for gestational week at sampling, maternal pre-pregnancy BMI, gestational weight gain, parity, employed and gestational diabetes mellitus.

SGA, small size for gestational age; LGA large size for gestational age; OR, odds ratio; CI, confidence interval; T3, triiodothyronine; T4, thyroxin; TSH, thyroid stimulating hormone; FT3, free triiodothyronine; FT4, free thyroxine.

Those highlighted in bold indicate that the associations showed statistical significance.

Stratified analyses by lower and higher vitamin A levels based on the median parameter (median = 0.46 mg/L).

## Discussion

To our knowledge, this is the first study to assess the effects of vitamin A on the relationships between thyroid hormones during early pregnancy and fetal growth outcomes in a prospective population. We observed that higher FT4 levels during early pregnancy showed protective effects for both SGA and LGA only among euthyroid women with higher vitamin A levels. These protective effects of FT4 on normal fetal growth have not been previously reported. The results also indicated that maternal FT3 levels were positively associated with birth weight among women with lower maternal vitamin A levels in early pregnancy. The human fetal thyroid gland synthesizes thyroid hormones after 12 weeks of gestation, and the fetus completely depends on maternal supply in the first trimester. Thyroid hormones and vitamin levels in early pregnancy are important for fetal development, so we chose the first trimester to assess the effect of vitamin A on the relationship between maternal thyroid hormones in early pregnancy and fetal growth. Our results will be useful in the application of evidence-based prenatal care quality improvement initiatives aimed at reducing adverse fetal growth outcomes by targeting screening thyroid function and essential nutrients in the first trimester.

Although FT4 was not associated with birth weight when modeled jointly with vitamin A (Table 3), increased levels of FT4 were associated with a significantly reduced risk of both SGA and LGA in the high vitamin A level group after full adjustment in the stratified analysis (Table 5). We also analyzed the association between FT4 and infant birth weight at different vitamin A levels in Supplementary Table 2. The results showed that FT4 levels were not significantly associated with infant birth weight in the different vitamin A level groups.

Our results are comparable to previous reports that use euthyroid pregnant women as in our study. Vrijkotte et al. found that increasing maternal FT4 in early pregnancy was associated with lower odds of LGA in male infants (23). Zhang et al. (10) evaluated 46,186 pregnant women during early and late pregnancy and reported that maternal low FT4 was associated with a higher risk of LGA neonates. A multicenter cohort study reported that the highest FT4 quintile among euthyroid women is not associated with adverse pregnancy outcomes, including SGA and preterm birth (24).

In pregnant women with subclinical thyroid dysfunction, some findings of research on the association of FT4 and fetal growth have also supported our results. A prospective cohort study from China found that isolated hypothyroxinemia (decreased FT4 with normal TSH) was associated with an increased risk for SGA in the first 20 weeks of pregnancy (25). Yuan et al. (26) reported that hyperthyroxinemia (increased FT4 with normal TSH) was associated with a decreased risk of LGA in late pregnancy. However, in the Ma'anshan birth cohort, maternal isolated hypothyroxinemia was not associated with an increased risk for SGA in either the first or the second trimester, and an increased risk of LGA was observed in the second trimester (27).

In our study, the protective effects of higher FT4 on SGA and LGA were simultaneously observed in euthyroid women with higher vitamin A levels in early pregnancy. This result indicated that vitamin A might act synergistically in cooperation with maternal FT4 on fetal growth. It is known that one of the most critical roles of vitamin A in human health is its effect on thyroid function. First, vitamin A interferes with iodine metabolism in the thyroid and regulates thyroid hormone metabolism. Vitamin A deficiency aggravates thyroid dysfunction caused by iodine-deficient diets (13). Second, vitamin A can modulate the effects of thyroid hormones on target tissues mediated by its metabolite RA. RA induces the expression of the thyroid hormone transporter and monocarboxylate transporter, which demonstrates cross-talk between RA signaling and thyroid hormone signaling in early development at the level of the thyroid hormone transporter (28). Although RAR and thyroid receptors do not seem to interact directly physically, they share some cofactors so that some form of competition may occur between the two ligands and their receptors (29). In conclusion, there are many levels at which vitamin A can interact with the physiology of the hypothalamo-pituitary-thyroid axis, which was also reflected by our findings, as significant positive correlations were detected with T3, FT3 and TSH, and negative correlations were detected with FT4. The associations suggest thyrotropic actions of vitamin A occurred not only at the level of the maternal thyroid but also at the level of the maternal pituitary. According to our findings, randomized intervention studies will be warranted to evaluate whether vitamin A-related differences will display significant clinical impact.

Our study found a positive association of FT3 and birth weight in pregnant women with lower vitamin A levels. FT3 is a principal bioactive thyroid hormone that directly exerts a biological effect on fetal growth. High FT3 concentrations act through anabolic effects on fetal metabolism and the stimulation of fetal oxygen consumption for the general accretion of the fetal mass from early gestation (30). Those women with low levels of vitamin A often coexist with lower iodine levels (15). In rat experiment, compared to the healthy group or diets deficient in iodine group, FT4 and T4 were lower in the diets deficient in vitamin A and iodine group, but there were no significant differences in T3 or FT3 concentrations among groups (31). The activity of FT3 was much higher than that of FT4. This regulation would explain the effect of FT3 on birth weight in conditions of lower vitamin A levels. Few studies have investigated the relationship between maternal FT3 in early pregnancy and fetal growth. A previous study from China showed that maternal higher FT3 within the normal range concentrations during early pregnancy was associated with an increased risk for low birth weight (OR = 2.52, 95% CI: 1.00, 6.36 per unit increase in FT3 concentrations) among normal weight women with inadequate gestational weight gain (32). The mechanism by which maternal FT3 positively affects birth weight at low vitamin A levels is unclear. Our observations should be considered hypothesis generating, and prospective investigations with larger sample sizes and mechanistic studies should be conducted in this field.

Vitamin A was not associated with any fetal growth outcomes in this cohort. Vitamin A was associated with fetal growth only when modeled jointly with thyroid hormones. Therefore, it is unlikely that the modifying effects are attributable to mediation through vitamin A.

The strengths of our study included the use of data from a prospective population-based cohort, which provided screening data of thyroid function and an important nutrient in the first trimester and information on potential confounders and mediating factors to help us better understand the association between thyroid function and fetal growth outcomes. A sufficient sample size with measurements of T3, T4, TSH, FT3, FT4 and vitamin A concentrations in early pregnancy allowed us to assess the interaction of maternal thyroid function and vitamin A levels and further evaluate whether the associations of thyroid function and fetal growth were modified by vitamin A. We adjusted for confounding to 6 important variables that were constant throughout all the models: maternal prepregnancy BMI, GWG, maternal employed, parity, gestational week at sampling, and GDM in data analysis to avoid overadjustment.

Limitations of the study included that we did not measure maternal iodine levels in urine, which have been associated with thyroid function and retinol and/or fetal growth (33, 34). Because the primary goal of this cohort study was not to evaluate effect of vitamin A on the relationship between maternal thyroid hormones and fetal growth, we did not collect maternal urine samples. It's impossible to rule out the possibility that some of the effects attributed to vitamin A are due to iodine. But the National Advocacy Programme for the Elimination of Iodine Deficiency Disorder (IDD) through salt iodization by 2000 was launched in China. Today IDD was eliminated at the national level and iodine nutrition of general population was maintained at an adequate level (35). Consequently, iodine insufficiency is likely to have a little impact on vitamin A effect estimations.

The recruitment was restricted to a single hospital in Beijing suburb, but two-thirds of the study population was not born in Beijing but came from all over the country. However, pregnant women in Beijing might have better health status than those in rural areas. Studies with wider populations are needed to clarify the associations.

Prenatal multivitamins intake is an important dietary exposure that is related to the vitamin A measure as well as to fetal growth outcomes. There were 64 women in the sample who had been taking multivitamins, including vitamin A, for 5.5 (2.6–9.6) weeks (median, IQR) before blood sample collection in early pregnancy. We compared the variables, including vitamin A levels, thyroid hormone levels, and birth outcomes, in the vitamin-taking group and the non-vitamin-taking group (Supplementary Table 3). Except for TSH (*p* = 0.042), the other variables were not significantly different between the two groups.

Our results showed that maternal FT4 in early pregnancy was positively associated with optimal fetal growth among women with higher serum vitamin A concentrations. This suggests that increased vitamin A intake during the first trimester of pregnancy might act synergistically in cooperation with maternal FT4 and have protective effects on fetal growth.

## Conclusion

We examined the effect modification of serum vitamin A on thyroid hormones in early pregnancy and fetal growth outcomes among euthyroid pregnant women. We observed the associations between higher FT4 levels and decreased odds of SGA and LGA, which were modified by whether a mother was at a higher vitamin A level. However, FT3 concentrations in relation to increased birth weight only occurred in women with lower vitamin A levels. Our findings suggest that vitamin A might act synergistically in cooperation with maternal thyroid hormones during early pregnancy to ensure normal fetal growth.

## Data availability statement

The original contributions presented in the study are included in the article/Supplementary material, further inquiries can be directed to the corresponding author.

## Ethics statement

The studies involving human participants were reviewed and approved by the Ethics Committees of the Capital Institute of Pediatrics (8 November 2016; Approval No. SHERLL-2016034). Written informed consent to participate in this study was provided by the participants' legal guardian/next of kin.

## Author contributions

YL designed the study, collected the data, and performed the statistical analysis. LW and YL wrote the manuscript. QX, HZ, GX, XC, and RM revised the manuscript. ZS collected the data and revised the manuscript. All authors contributed to the article and approved the submitted version.

## Funding

This study was supported by the Directional Guidance Fund of Capital Institute of Pediatrics (Grant No. FX-15-04).

## Conflict of interest

The authors declare that the research was conducted in the absence of any commercial or financial relationships that could be construed as a potential conflict of interest.

## Publisher's note

All claims expressed in this article are solely those of the authors and do not necessarily represent those of their affiliated organizations, or those of the publisher, the editors and the reviewers. Any product that may be evaluated in this article, or claim that may be made by its manufacturer, is not guaranteed or endorsed by the publisher.
