# Supplementary material for: Effect of vitamin A on the relationship between maternal thyroid hormones in early pregnancy and fetal growth: A prospective cohort study
Source: Front Nutr. 2022 Aug 24;9:980853. doi: 10.3389/fnut.2022.980853 (PMC9449534; doi:10.3389/fnut.2022.980853)
Supplement: Supplementary file 1 [file Data_Sheet_1.pdf]

Supplementary Table 1 Pearson correlations between individuals for maternal thyroid hormones and vitamin A levels measured in early pregnancy

	Vitamin A	T3	T4	TSH #	FT3
T3	0.22571***				
T4	0.03417	0.53307***			
TSH #	0.10079*	-0.285***	-0.42105***		
FT3	0.10852**	0.48328***	0.26879***	-0.20776***	
FT4	-0.21629***	0.12848**	0.4101***	-0.45799***	0.35515***

#Natural log transformed

*P<0.05

**P<0.001

***P<0.0001

T3, triiodothyronine; T4, thyroxin; TSH, thyroid stimulating hormone; FT3, free triiodothyronine; FT4, free thyroxine.

Supplementary Table 2 Associations between maternal FT4 and infant birth weight, stratified by vitamin A level.

FT4	Lower vitamin A level (n = 319)		Higher vitamin A level (n = 318)	
	β (95% CI)	p-value	β (95% CI)	p-value
Model 1[a]	0.011 (-0.010-0.032)	0.314	-0.004 (-0.029-0.022)	0.784
Model 2[b]	0.021 (-0.001-0.042)	0.059	0.001 (-0.024-0.025)	0.974

[a] Model 1: Adjusted for gestational week at sampling, fetal sex, and gestational age at birth (linear and quadratic).

[b] Model 2: Adjusted for model 1 plus maternal pre-pregnancy BMI, parity, employed, gestational weight gain, gestational diabetes mellitus.

FT3, free triiodothyronine; CI, confidence interval.

Stratified analyses by lower and higher vitamin A levels based on the median parameter (median = 0.46).

Supplementary Table 3 Distribution of maternal thyroid hormones, vitamin A levels in early pregnancy, and birth outcomes in the vitamin-taking group and the nonvitamin-taking group.

	Vitamin-taking group (n = 64)	Nonvitamin-taking group (n = 573)	p-value
	Median (IQR) or N (%)	Median (IQR) or N (%)	
Vitamin A (mg/L)	0.49 (0.42-0.56)	0.46 (0.41-0.53)	0.051
T3 (nmol/L)	1.98 (1.63-2.17)	1.89 (1.67-2.16)	0.321
T4 (nmol/L)	133.34 (118.54-146.38)	128.17 (113.63-144.46)	0.069
TSH (mIU/L)	1.07 (0.57-1.62)	1.25 (0.72-1.84)	**0.042**
FT3 (pmol/L)	4.87 (4.68-5.45)	4.98 (4.68-5.41)	0.239
FT4 (pmol/L)	10.91 (9.73-12.30)	10.91 (9.95-11.85)	0.435
SGA	4 (6.25)	33 (5.76)	0.479
AGA	55 (85.94)	515 (89.88)	0.479
LGA	5 (7.81)	25 (4.36)	1.576

SGA, small size for gestational age; AGA, average size for gestational age; LGA, large size for gestational age; IQR, interquartile range; T3, triiodothyronine; T4, thyroxin; TSH, thyroid stimulating hormone; FT3, free triiodothyronine; FT4, free thyroxine.

Those highlighted in bold indicate that the associations showed statistical significance.
